# The role of mediator subunit MED7 in Arabidopsis development

**DOI:** 10.3389/fpls.2025.1542950

**Published:** 2025-03-07

**Authors:** Koppolu Raja Rajesh Kumar, Jeanette Blomberg, Stefan Björklund

**Affiliations:** ^1^ Department of Biotechnology, Indira Gandhi National Tribal University, Amarkantak, India; ^2^ Department of Medical Biochemistry and Biophysics, Umeå University, Umeå, Sweden

**Keywords:** mediator, *Arabidopsis thaliana*, MED7, hyponastic cotyledons, elongated hypocotyls, modified root architecture

## Abstract

MED7, a middle-module subunit of the transcriptional co-regulator Mediator complex, plays a critical role in gene regulation in *Arabidopsis thaliana*, where it is encoded by two paralogs, *MED7A* and *MED7B*. We present phenotypic analyses of homozygous MED7-silenced transgenic lines with significantly reduced expression of both *MED7* paralogs under autotrophic conditions. Our findings demonstrate that MED7 is essential for proper cotyledon opening during de-etiolation, as the silenced lines showed a marked delay in this process. Additionally, these lines displayed distinct morphological alterations, including hyponastic cotyledons, elongated hypocotyls, and modified root architecture, such as shorter primary roots and impaired root hair development in light-grown seedlings. *MED7* silencing also significantly hindered light-induced adventitious root (AR) formation on the hypocotyls of etiolated seedlings, leading to a notable reduction in AR production. Moreover, *MED7* silencing impacted the timing of floral transition and shoot branching, resulting in delayed flowering and an increased number of primary cauline branches on the inflorescence stem. Together, these results underscore a central role for MED7 in orchestrating key developmental processes in plants.

## Introduction

Mediator is a highly conserved and essential protein complex that plays a central role in transcriptional regulation across all eukaryotes (Soutourina, 2018). As a key component of the transcriptional machinery, Mediator functions as a critical co-regulator, bridging gene-specific transcription factors (TFs) with the RNA polymerase II (Pol II) pre-initiation complex. By receiving and integrating regulatory information from promoter-bound TFs and enhancers, Mediator modulates transcription across nearly all protein-coding genes in eukaryotes (Allen and Taatjes, 2015). Beyond initiation, Mediator contributes to multiple other regulatory processes involved in gene expression, including chromatin looping, mRNA processing and export, transcriptional memory, and re-initiation (Reeves and Hahn, 2003; D’Urso et al., 2016; Chereji et al., 2017; Crawford et al., 2020; Ramasamy et al., 2023).

Mediator comprises 25 to 35 subunits, varying by organism, and is organized into distinct modules: head, middle, tail (core Mediator), and a cyclin-kinase module (CKM), which associates transiently with core Mediator (Elmlund et al., 2006). Head and middle interact with Pol II, including its C-terminal domain (CTD), as well as with general transcription factors (GTFs), while tail binds to sequence-specific TFs at promoters or enhancers (Warfield et al., 2022). Although Mediator has been extensively studied in yeast and metazoans, research on plant Mediator is more recent. Similar to its role in other eukaryotes, plant Mediator serves as a central hub for transcriptional regulation, coordinating gene expression for development, metabolism, and responses to hormones and stress (Buendía-Monreal and Gillmor, 2016; Sinha and Kumar, 2021; Blomberg et al., 2024). However, plants have evolved unique subunits, likely facilitating interactions with plant-specific TFs and enabling the integration of signals related to hormonal responses, pathogen defense, and stress adaptation (Bäckström et al., 2007; Dolan and Chapple, 2016).

In plants Mediator plays a pivotal role in regulating responses to key hormones such as auxins, gibberellins, and abscisic acid, as well as in the transcriptional activation of genes within hormonal pathways, which are essential for plant growth and development (Buendía-Monreal and Gillmor, 2016; Kumar et al., 2018). Beyond its central role in transcriptional regulation, plant Mediator acts as a crucial integrator of diverse signaling pathways, enabling plants to effectively respond and adapt to dynamic environmental conditions (Dolan and Chapple, 2016). Studies using loss-of-function mutants and silenced lines of various subunits have shed light on their distinct roles in plant development and environmental responses. For instance, a *MED18* loss-of-function mutant exhibits delayed flowering and altered floral organ development (Zheng et al., 2013) while MED8 is essential for organ size regulation; mutants lacking *MED8* develop smaller flowers due to reduced cell expansion (Xu and Li, 2012). *MED30* is critical for early development, particularly embryogenesis, and reduced MED30 expression delays flowering (Jaskolowski et al., 2019). Furthermore, MED25 has been shown to regulate lateral root formation (Raya-González et al., 2014).

The Arabidopsis MED7 subunit is part of the Mediator middle module and is encoded by two paralogs, *MED7A* and *MED7B*. Studies in various organisms, including plants and fungi, highlight MED7’s importance in diverse biological processes such as growth, development, and responses to environmental stresses (Tebbji et al., 2014; Kumar et al., 2018). However, research on MED7’s specific functions in plants remains limited. In previous work, we demonstrated the redundant roles of MED7A and MED7B in regulating skotomorphogenic growth in Arabidopsis seedlings. Our findings showed that MED7 influences expression of genes involved in cell elongation and response to auxin and brassinosteroids processes essential for etiolated seedling growth under dark conditions. Additionally, concurrent silencing of MED7A and MED7B (hereafter referred to as *med7RI* lines) resulted in shorter hypocotyls and defective hook opening in dark-grown seedlings, underscoring MED7’s critical role in early seedling development during skotomorphogenesis (Kumar et al., 2018).

In this study, we investigate the effects of *MED7A*/*MED7B* silencing across multiple stages of Arabidopsis development, revealing a broader impact of MED7 on plant growth and developmental processes beyond skotomorphogenesis.

## Materials and methods

### Plant materials, growth conditions and treatments

In this study, we used *Arabidopsis thaliana* ecotype Columbia (Col-0) as the wild-type (WT) control. Two previously characterized homozygous *MED7A*/*MED7B* silenced transgenic lines, *med7RI-7* and *med7RI-13*, with significantly reduced expression of both *MED7* paralogs, were included (Kumar et al., 2018). A homozygous empty vector (EV) line was also used as a control alongside WT plants (Kumar et al., 2018). Additionally, we employed *med7RI* lines conditionally complemented with epitope-tagged MED7A (FLAG-MED7AR/*med7RI*) and MED7B (HA-MED7BR/*med7RI*), each expressed individually under an estradiol-inducible promoter (cloned into the pER8 vector), along with a control complementation line (empty pER8 vector)/*med7RI*) as previously described (Kumar et al., 2018). Seeds from WT and transgenic lines were surface-sterilized with 70% ethanol containing 0.1% Tween 20, followed by two quick washes in 95% ethanol. After drying, seeds were plated on half-strength Murashige and Skoog (½ × MS, pH 5.7) basal salt medium (Sigma-Aldrich, Stockholm, Sweden) containing 0.8% (w/v) phytoagar (Duchefa Biochemie). The plated seeds were stratified at 4°C in darkness for three days to synchronize germination. After stratification, seeds were transferred to photoperiodic light conditions (120-130 µmol m^−2^s^−1^) with a 16-hour light/8-hour dark cycle at 22°C and grown vertically. For etiolated seedling growth, stratified seeds were exposed to light for six hours to initiate germination, then shifted to complete darkness in a vertical orientation and grown at 22°C. Conditional expression of FLAG-MED7AR and HA-MED7BR was induced by growing the respective transgenic seedlings on medium containing 1.0 μM β-estradiol (Sigma-Aldrich) (Kumar et al., 2018).

### Cotyledon opening during de-etiolation

A qualitative analysis of cotyledon opening and expansion during de-etiolation was conducted using four-day-old etiolated seedlings. To induce de-etiolation, seedlings grown in darkness were exposed to continuous low-fluence white light at an intensity of 10 µmol m^-2^s^-1^ for 48 hours in a growth chamber (Percival Scientific, Iowa, USA). Cotyledon opening was monitored at regular intervals and observed under a dissecting microscope. Photographs were taken after 36 hours of light exposure to assess the progression of de-etiolation.

### Measurement of hypocotyl and primary root length

Seedlings were grown under photoperiodic light conditions (120–130 µmol m^-2^s^-1^, 16-hour light/8-hour dark cycle) for 10 days. They were then placed on a flat surface, and images were captured using a high-resolution camera. Hypocotyl and primary root lengths were measured using ImageJ (https://imagej.net/ij/). Hypocotyl length was measured from the base of the cotyledons to the root-hypocotyl junction, while primary root length was measured from the root-hypocotyl junction to the root tip.

### Quantification of adventitious roots

Four-day-old etiolated seedlings grown in darkness on ½× MS basal salt medium (pH 5.7) containing 0.8% (w/v) phytoagar were transferred to light conditions (120–130 µmol m^-2^s^-1^). After 7 days in light, photographs were taken, and ARs on the hypocotyls were counted.

### Flowering time and phenotypic analysis

Seeds were germinated and plants were grown under long-day conditions (16-hour light/8-hour dark photoperiod; 120–130 µmol m^-2^s^-1^, 22°C) using cool fluorescent lighting. In each experiment, at least 15 plants per genotype were analyzed, with tray positions randomized to minimize positional effects within the growth chamber. Flowering time was recorded as both the total number of rosette leaves at bolting and the number of days from sowing until bolting, defined as the visible emergence of the inflorescence stem above the rosette. At the mature flowering stage, the number of primary cauline branches (arising from the main inflorescence stem) and primary rosette branches were also counted.

### RNA isolation and RT-qPCR

Total RNA was extracted from both four-day-old old dark grown etiolated seedlings and from etiolated seedlings after 6 h exposure to white light at an intensity of 120–130 µmol m^-2^s^-1^ using the Omega Bio tek plant RNA kit and treated with RNase-free ezDNase following the manufacturer’s instructions (Thermo Scientific, USA). 1 μg of total RNA was reverse transcribed using iScript reverse transcription supermix (Biorad, Solna, Sweden). RT-qPCR was performed using a CFX96 Touch qPCR instrument (BioRAD USA) and the PowerUp SYBR green master mix (Applied Biosystems, Massachusetts, USA). Gene expression levels were normalized to the AT3G18780 and AT4G36800 reference genes and displayed in relative units. Three biological and two technical repetitions were performed for each sample. The sequences of the RT-qPCR primers for HY5 were described previously (Kumar et al., 2018). The sequences for PIF4 were; Forward: 5´-CCGACCGGTTTGCTAGATACATCG-3´, Reverse: 5´-ATCTCCATCGGCTGCATCTGAGTC-3´.

### Statistical analyses

Significance levels were assessed using Student´s t-test or one-way ANOVA, followed by mean comparisons with Tukey’s and Bonferroni’s *post hoc* tests. All statistical analyses were performed using Excel or the Origin software (https://www.originlab.com/).

## Results and discussion

### *MED7* silencing causes delayed opening of cotyledons during de-etiolation

Cotyledon opening in Arabidopsis is a key process in photomorphogenesis, the developmental transition triggered by light exposure. When etiolated seedlings are exposed to light, they undergo de-etiolation, which includes the expansion and opening of the cotyledons. This is crucial for shifting from heterotrophic to autotrophic growth, enabling seedlings to optimize light capture for photosynthesis. Brassinazole (BRZ), a brassinosteroid (BR) biosynthesis inhibitor, can induce photomorphogenic traits such as short hypocotyls and cotyledon opening in dark-grown Arabidopsis seedlings. In a previous study, we observed that the unfolding of cotyledons in response to BRZ treatment was significantly impaired in the *med7RI* lines, indicating that MED7 silencing impacts not only skotomorphogenesis but also photomorphogenesis in Arabidopsis (Kumar et al., 2018).

**Figure 1:**
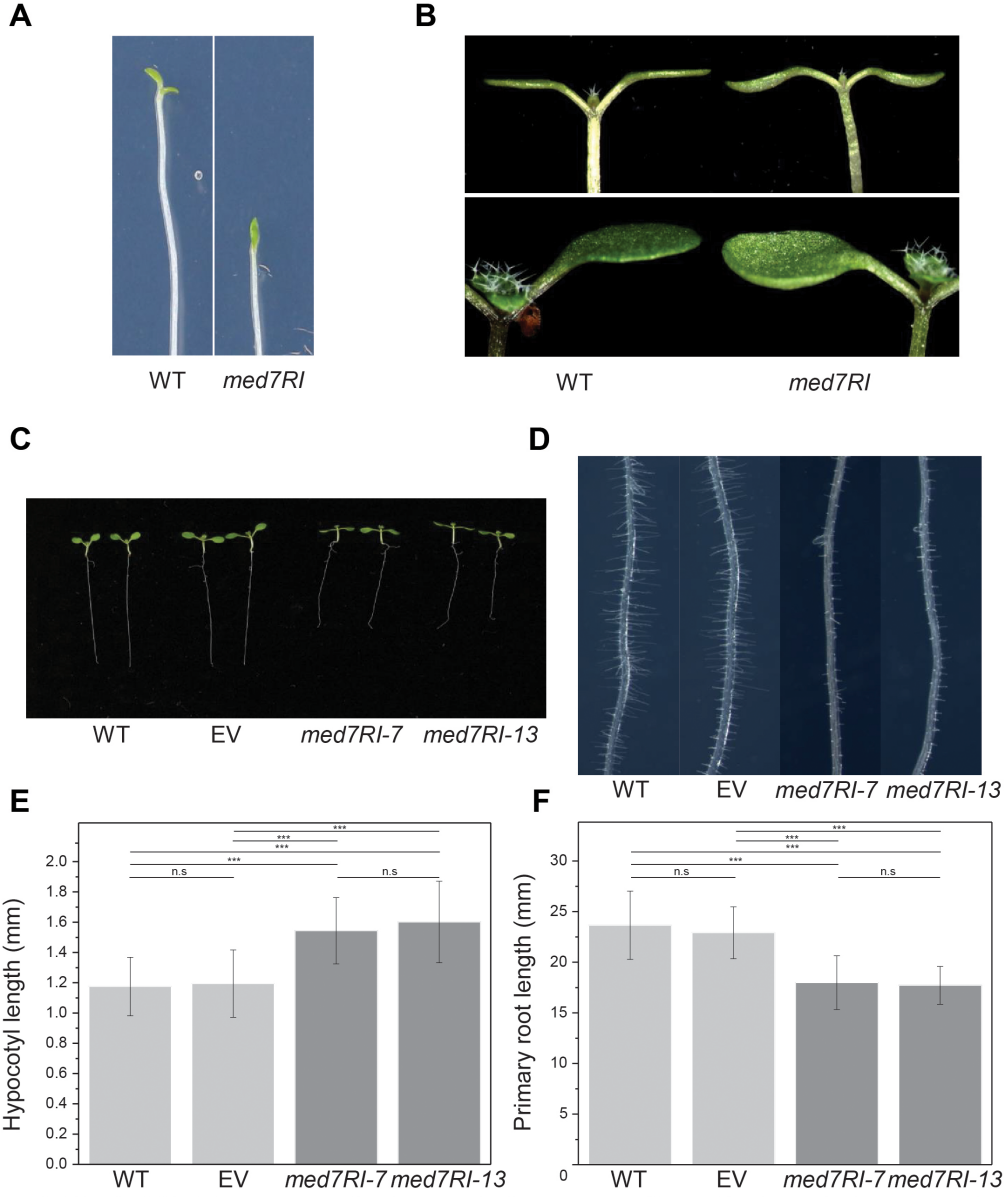
Effect of *MED7* silencing on light-grown Arabidopsis seedlings. **(A)** Delayed cotyledon opening during de-etiolation. Four-day-old etiolated seedlings were exposed to 10 μE white light, and images were captured after 36 hours. Representative images of wild-type (WT) and *med7RI* line is shown. **(B)** Hyponastic cotyledon phenotype in *MED7*-silenced lines compared to control lines. Images of WT and *med7RI* seedlings are presented. **(C)** Ten-day-old seedlings of control (WT, EV) and *med7RI* (*med7RI-7, med7RI-13*) lines, displaying differences in growth patterns under photoperiodic light. **(D)** Impaired root hair development in *MED7*-silenced lines. Root hair growth on the primary root of ten-day-old seedlings from control (WT, EV) and *med7RI* lines is shown. **(E)** Hypocotyl length and **(F)** primary root length of ten-day-old light-grown seedlings from control and *med7RI* lines. Data represent mean ± SD, with significance indicated by asterisks (***P < 0.001). Not statistically significant differences are labelled n.s. The experiments were performed using three biological replicates, with ≥30 seedlings per line/genotype, and statistical significance was determined using one-way ANOVA, followed by Tukey’s and Bonferroni’s *post hoc* tests.

To further investigate the impact of MED7 silencing on cotyledon opening during de-etiolation, we monitored cotyledon opening under low-fluence continuous white light. The *med7RI* lines exhibited a delayed response in cotyledon opening compared to the control lines. After 36 hours of exposure to continuous low-fluence white light, control seedlings (WT and EV) showed fully opened cotyledons, while the *med7RI* lines remained closed (**Figure 1A**, **Supplementary Figure 1A**). However, by 48 hours, most *med7RI* seedlings had initiated cotyledon opening, indicating that the opening and expansion of cotyledons were delayed in the *med7RI* lines compared to the controls.

The delayed cotyledon opening in *med7RI* lines during de-etiolation highlights the importance of MED7 in regulating light signaling pathways that facilitate the transition from skotomorphogenesis to photomorphogenesis. ELONGATED HYPOCOTYL 5 (HY5) and PHYTOCHROME INTERACTING FACTORs (PIFs) are transcription factors that function as master regulators that control a large array of genes involved in light-dependent development. PIFs and HY5 are oppositely regulated by light conditions and therefore have contrasting effects on light-induced cotyledon opening in Arabidopsis (Zhang et al., 2017; Yao et al., 2024). In darkness, CONSTITUTIVE PHOTOMORPHOGENIC 1 (COP1) degrades HY5, relieving repression on elongation genes and enabling PIFs to accumulate and activate hypocotyl elongation pathways (Yu et al., 2013). Upon light exposure, phytochromes phosphorylate PIFs, marking them for ubiquitin-mediated degradation, which suppresses elongation and promotes photomorphogenesis (Jia et al., 2014). In light-grown seedlings, HY5 promotes inhibition of hypocotyl elongation by activating photomorphogenesis-associated genes, repressing elongation-related genes through chromatin modifications via its interaction with HDA15, and enhancing BIN2 kinase activity to suppress brassinosteroid signaling (Xu et al., 2016; Zhao et al., 2019; Li et al., 2020). We have previously reported that *HY5* levels are significantly lower in etiolated *med7RI* seedlings grown in dark compared to wild-type (Kumar et al., 2018). We used RT-qPCR to analyze the *HY5* mRNA levels in etiolated seedlings before and after 6 hours of exposure to light. We found that the expression levels of *HY5* were reduced in the *med7RI* lines both at skotomorphogenic and during de-etiolation compared to control lines (**Supplementary Figure 1B**). On the other hand, PIF4 levels were relatively higher in *med7RI* lines compared to control lines in both dark and light conditions. This indicates that defects in expression of HY5 and PIF4 during de-etiolation contribute to the delayed cotyledon opening observed in *med7Ri* lines.

### Impact of *MED7* silencing on cotyledon morphology

We observed that *med7RI* lines display hyponastic cotyledons, characterized by an upward bending of the cotyledons (**Figure 1B**). This was reversed upon expression of either Flag-MED7aR or HA-MED7bR (**Supplementary Figure 2A**). This morphological change appears to result from disruptions in the normal growth patterns and hormonal signaling pathways that are essential for proper cotyledon development and orientation. Importantly, hyponasty was restricted to cotyledons and was not observed in the true leaves of *med7RI* lines.

Auxin, a hormone essential to nearly all aspects of plant development, including postembryonic organogenesis, has been linked to similar phenotypes. For example, hyponastic cotyledons are observed in the *rol1-2* mutant, where altered flavonol profiles lead to significant changes in auxin transport and distribution, marked by a basal-to-apical shift in the polar localization of PIN2, a key auxin efflux carrier (Kuhn et al., 2017). Previously, we reported that several *AUX/IAA* family genes and other auxin-responsive genes are differentially regulated in etiolated *med7RI* seedlings (Kumar et al., 2018), suggesting that disruptions in auxin homeostasis may contribute to the hyponastic cotyledons observed in *med7RI* lines. Interestingly, mutations in *CDK8*, encoding a subunit of the Mediator kinase module, reversed the hyponastic cotyledons in *rol1-2* mutants to an epinastic state (Schumacher et al., 2021).

### Hypocotyl and root characteristics in light-grown *med7RI* seedlings

To examine the role of MED7 in light-grown conditions, we compared hypocotyl and primary root lengths in *med7RI* lines with WT and EV plants. In contrast to the reduced hypocotyl length seen in etiolated seedlings with *MED7* silencing (Kumar et al., 2018), hypocotyls of light-grown *med7RI* seedlings were significantly longer than those of control lines (**Figures 1C, E**). Additionally, the primary root length in *med7RI* lines was markedly shorter than in WT (**Figures 1C, F**). Expression of either Flag-MED7aR or HA-MED7bR reversed both phenotypes (**Supplementary Figures 2B–D**). Furthermore, *med7RI* lines exhibited abnormal root hair development along the primary root, with predominantly short and unevenly distributed root hairs compared to controls (**Figure 1D**). However, no significant changes in lateral root density were observed between the control and *med7RI* lines (**Supplementary Figure 2E**).

The contrasting effects of *MED7* silencing on hypocotyl elongation under dark and light conditions underscore the complexity of its role in development. In etiolated seedlings, *MED7* silencing results in shorter hypocotyls due to impaired cell elongation, likely caused by altered expression of genes involved in cell wall remodeling and hormone signaling, particularly pathways involving auxin and brassinosteroids (Kumar et al., 2018). Conversely, in light-grown seedlings, *MED7* silencing leads to increased hypocotyl length, potentially due to differential regulation of light-responsive genes and hormonal pathways that promote elongation in response to light (Vandenbussche et al., 2005). It is likely that the increased hypocotyl elongation in light-grown *med7RI* seedlings is associated with defects in light-signaling pathways mediated by the dysregulation of key regulators, such as PIF4 and HY5 whose expression levels were affected in *med7RI* lines (**Supplementary Figures 1B, C**v). Both HY5 and PIF4 are crucial in controlling hypocotyl growth in response to light, with HY5 and PIF protein stability being oppositely regulated by light conditions (Zhang et al., 2017).

The reduced primary root length in light-grown *med7RI* lines suggests that MED7 is also involved in root development under light conditions. Light signaling is known to coordinate shoot and root development, where TFs like HY5, PIFs and various hormonal pathways mediate crosstalk between organs (Chen et al., 2016; Yang and Liu, 2020). Our current and previous findings that *HY5* expression is downregulated both during skotomorphogenic and de-etiolation in the *med7RI* lines may therefore account for their altered root growth, supporting the role of MED7 in light-dependent developmental processes (Kumar et al., 2018). These findings indicate that MED7 interacts with or regulates distinct factors in skotomorphogenic versus photomorphogenic conditions, allowing the plant to adapt root and shoot growth in response to light.

Previous studies have shown that auxin homeostasis and distribution are essential for proper root hair development, with auxin transporters like PIN proteins playing a key role in establishing the auxin gradients necessary for root hair elongation (Rigas et al., 2013). Similar root hair phenotypes have been observed in the *rol1-2* mutant, where disrupted auxin homeostasis results in shorter root hairs and a reduced primary root length (Schumacher et al., 2021). Additionally, shorter root hairs have been reported in other Mediator subunit mutants, such as *med12* and *med13*, which also affect auxin response pathways (Raya-González et al., 2014). Our findings suggest that *MED7* silencing may disrupt auxin distribution or signaling, contributing to the shorter root and root hair phenotypes observed in the *med7RI* lines.

### The *med7RI* lines display impaired AR development

**Figure 2:**
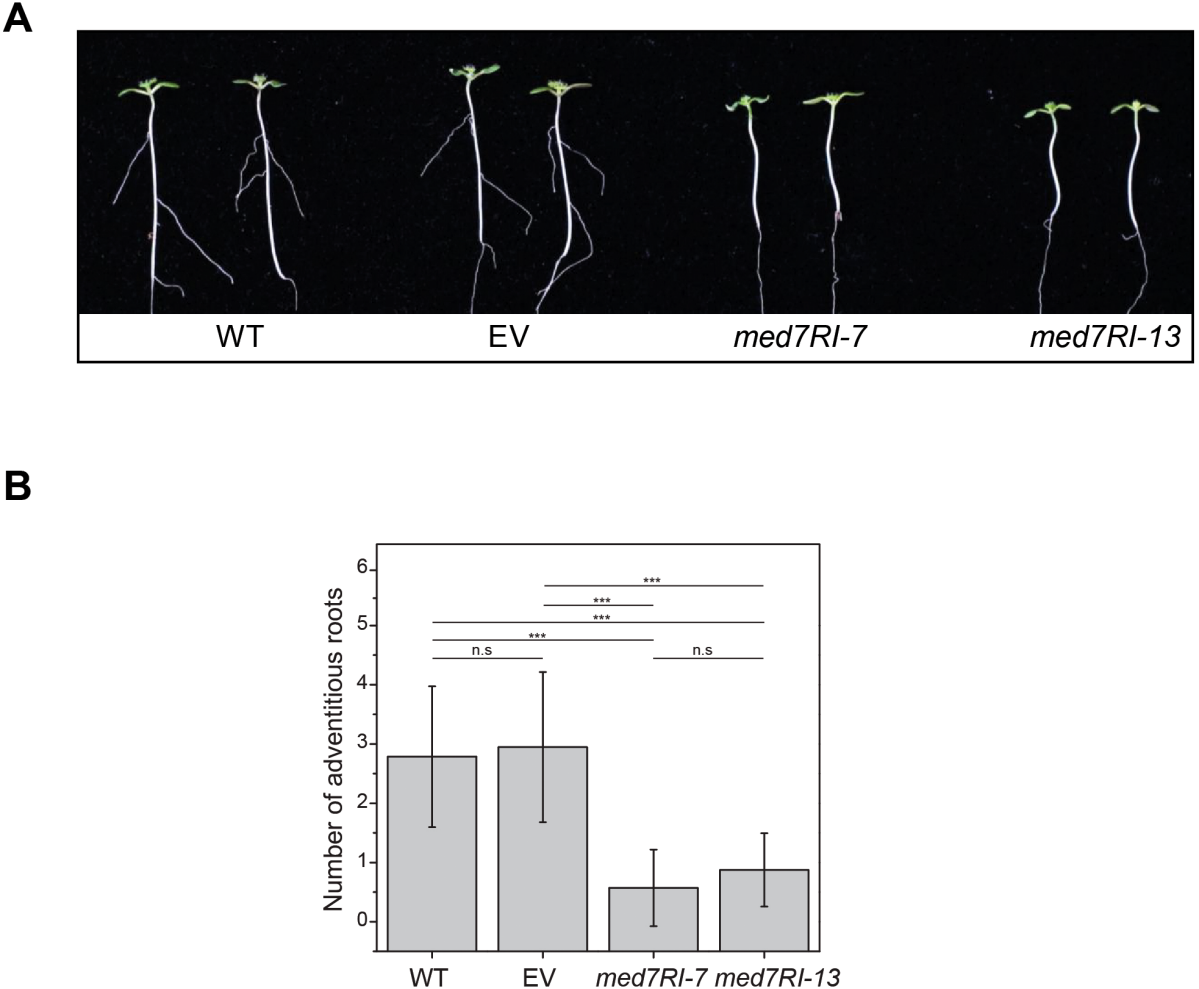
Effect of *MED7* silencing on AR development in Arabidopsis. **(A)** Four-day-old etiolated seedlings grown on 1/2 x MS medium were exposed to light to induce AR formation along the hypocotyl. After 7 days of light exposure, AR development was observed, showing a marked reduction in *MED7*-silenced lines (*med7RI-7* and *med7R1-13*) compared to controls (WT, EV). Representative images are shown. **(B)** The number of ARs was quantified after 7 days of light exposure in both control and *med7RI* lines. Results demonstrate a significant decrease in AR numbers in *MED7*-silenced lines. Data represent the mean ± SD, with significance indicated by asterisks (***P < 0.001). Not statistically significant differences are labelled n.s. The experiments were performed using three biological replicates, with ≥30 seedlings per each line/genotype, and statistical significance was determined using one-way ANOVA, followed by Tukey’s and Bonferroni’s *post hoc* tests.

In Arabidopsis, AR development along the hypocotyl is a well-documented response when etiolated seedlings are exposed to light. This process is primarily regulated by auxin signaling pathways and other factors that influence hypocotyl growth during the transition from etiolation to de-etiolation. To assess the impact of *MED7* silencing on AR development, we examined AR induction in *med7RI* seedlings. Unlike the WT and EV control lines, *med7RI* seedlings showed a nearly complete absence of AR formation after 7 days of light exposure (**Figures 2A, B**). In Arabidopsis, AR development is predominantly controlled by auxin homeostasis, transport, and signaling, along with interactions with other hormones and environmental cues, such as light (Pacurar et al., 2014; Zeng et al., 2022). Auxin-driven AR formation is mediated by AUXIN RESPONSE FACTORS (ARFs), which regulate the expression of genes involved in root initiation. ARF6 and ARF8 positively regulate AR formation, whereas ARF17 acts as a negative regulator (Gutierrez et al., 2009). Furthermore, auxin transport via PIN and AUX/LAX transporters is essential for AR development in Arabidopsis hypocotyls (Da Costa et al., 2020). The observed reduction in AR formation in *med7RI* lines may be linked to the previously documented defects in hypocotyl growth and auxin-responsive gene expression in these lines (Kumar et al., 2018). This suggests that the MED7 subunit is critical not only for proper etiolation and de-etiolation processes but also for maintaining auxin homeostasis—key factors for normal AR development. The absence of well-developed ARs in *med7RI* seedlings highlights the importance of MED7 in regulating pathways essential for AR formation. Furthermore, both *MED7* paralogs successfully restored AR development in the complementation lines (**Supplementary Figure 2F**).

### MED7 is required for timely flowering and normal branching

**Figure 3:**
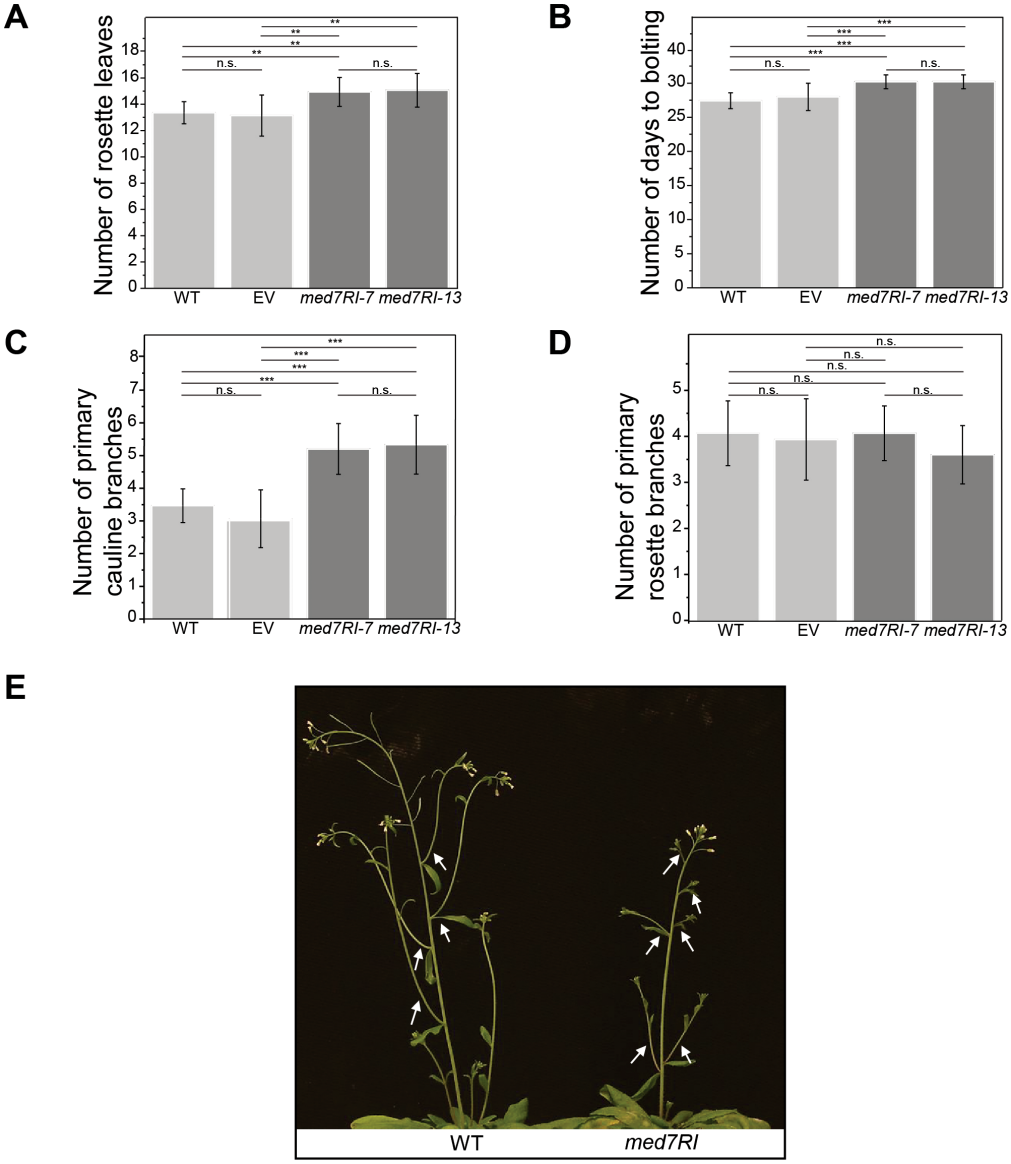
Effects of *MED7* silencing on flowering time and branching in Arabidopsis. Plants were grown under long-day conditions (16 h light/8 h dark). **(A)** Number of rosette leaves: Flowering time was assessed by recording the number of rosette leaves at bolting. *MED7*-silenced lines (*med7RI-7, med7RI-13*) exhibited a greater number of rosette leaves, indicating delayed flowering compared to controls (WT, EV). **(B)** Days to bolting: The number of days from sowing to bolting was recorded, showing a significant delay in flowering in *MED7*-silenced lines compared to control lines. **(C)** Primary cauline branches: *MED7*-silenced lines displayed a significant increase in the number of primary cauline branches, suggesting altered shoot branching. **(D)** Primary rosette branches: The number of primary branches emerging from the rosette was analyzed, comparing control and *med7* lines. **(E)** A comparison of WT and *med7RI* plants, with arrows indicating the increased number of primary cauline branches in *MED7*-silenced lines. Data represent the mean ± SD. Asterisks indicate the level of significance (***P < 0.001, **P < 0.01). Not statistically significant differences are labelled n.s. The experiments were performed using three biological replicates, with 15 plants per each line/genotype, and statistical significance was determined using one-way ANOVA, followed by Tukey’s and Bonferroni’s *post hoc* tests.

Following our assessment of developmental phenotypes in *med7RI* lines during the seedling stage, we evaluated floral transition in the adult plants. In *med7RI* lines, flowering was significantly delayed, as indicated by an increased number of rosette leaves and a longer time to bolting (**Figures 3A, B**). Furthermore, *med7RI* plants displayed a substantial increase in the number of primary cauline branches (**Figures 3C, E**), while the number of rosette branches remained normal (**Figure 3D**).

In *Arabidopsis thaliana*, flowering is controlled by key genes like *FLOWERING LOCUS T* (*FT*), which promotes flowering, and *FLOWERING LOCUS C* (*FLC*), which acts as a repressor. These genes integrate environmental signals, including photoperiod and vernalization, to optimize the timing of flowering (Searle et al., 2006). Additionally, multiple Mediator subunits play crucial roles in floral transition by modulating the expression of these and other flowering-related genes (Buendía-Monreal and Gillmor, 2016). Auxin also significantly influences branching by modulating levels of phytohormones like strigolactones and cytokinins, which collectively shape shoot architecture and branching patterns (Fichtner et al., 2022). In this study, the delayed flowering and increased primary cauline branching observed in *med7RI* lines suggest disruptions in either the regulation of flowering via *FT* and *FLC* or in the auxin and associated phytohormone pathways that control branching.

## Conclusion

Mediator acts as a central regulator of gene expression, integrating diverse environmental and developmental signals to ensure coordinated plant growth and adaptation. The delayed cotyledon opening and impaired AR development observed with *MED7* silencing highlight *MED7*’s key role in light- and auxin-regulated processes in Arabidopsis development. Additionally, the delays in flowering and increased cauline branching underscore MED7’s significance in orchestrating major developmental transitions. Together, these findings position MED7 as essential for multiple developmental processes. Further investigation into the precise mechanisms through which MED7 modulates these pathways will yield deeper insights into the complex regulation of plant growth and development.

## Data Availability

The original contributions presented in the study are included in the article/**Supplementary Material**. Further inquiries can be directed to the corresponding author.
