# Supplementary figures and images for: The role of mediator subunit MED7 in Arabidopsis development
Source: Front Plant Sci. 2025 Mar 7;16:1542950. doi: 10.3389/fpls.2025.1542950 (PMC11925930; doi:10.3389/fpls.2025.1542950)

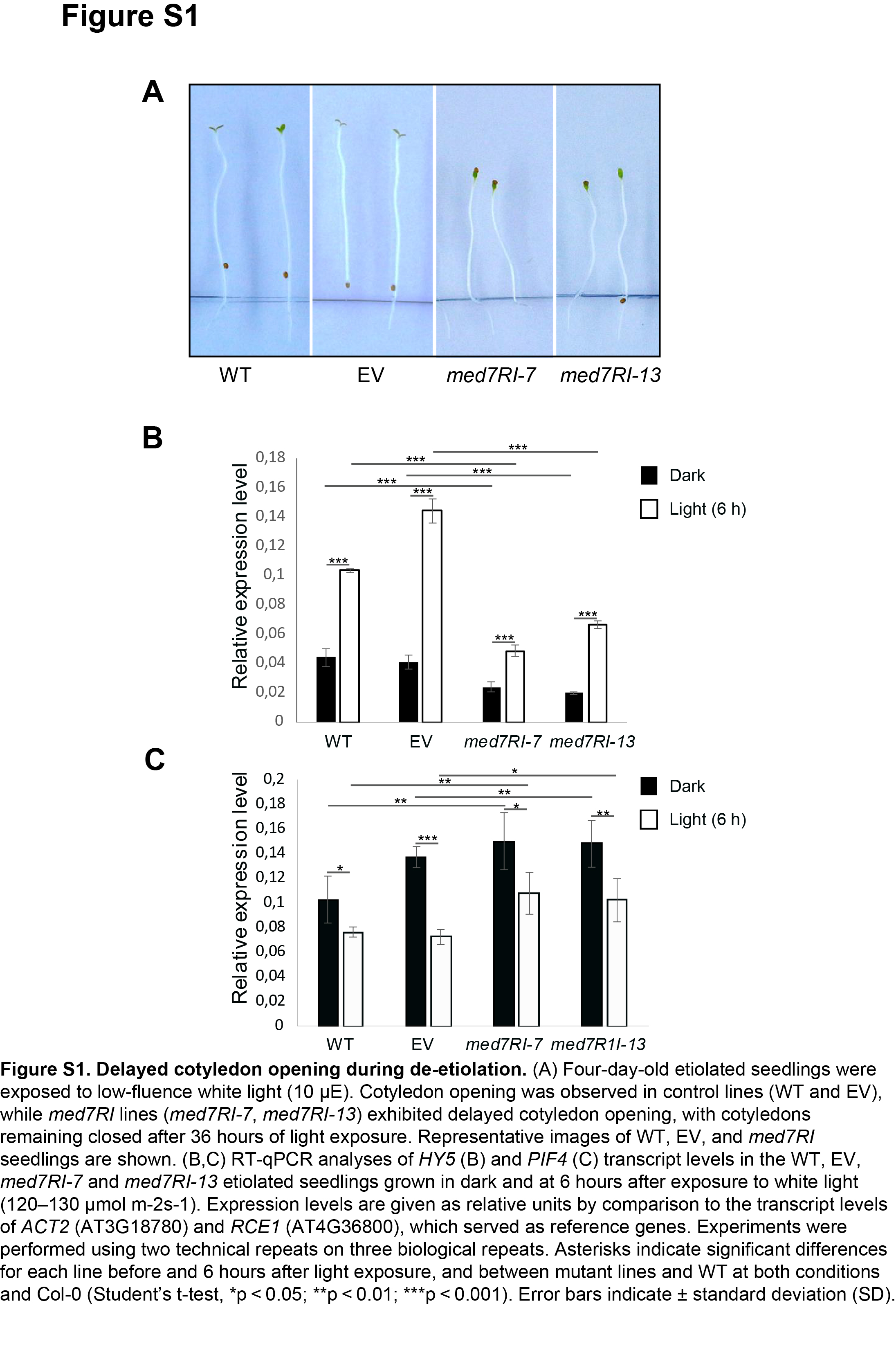

Supplement: Supplementary file 1 [file Image1.jpeg]

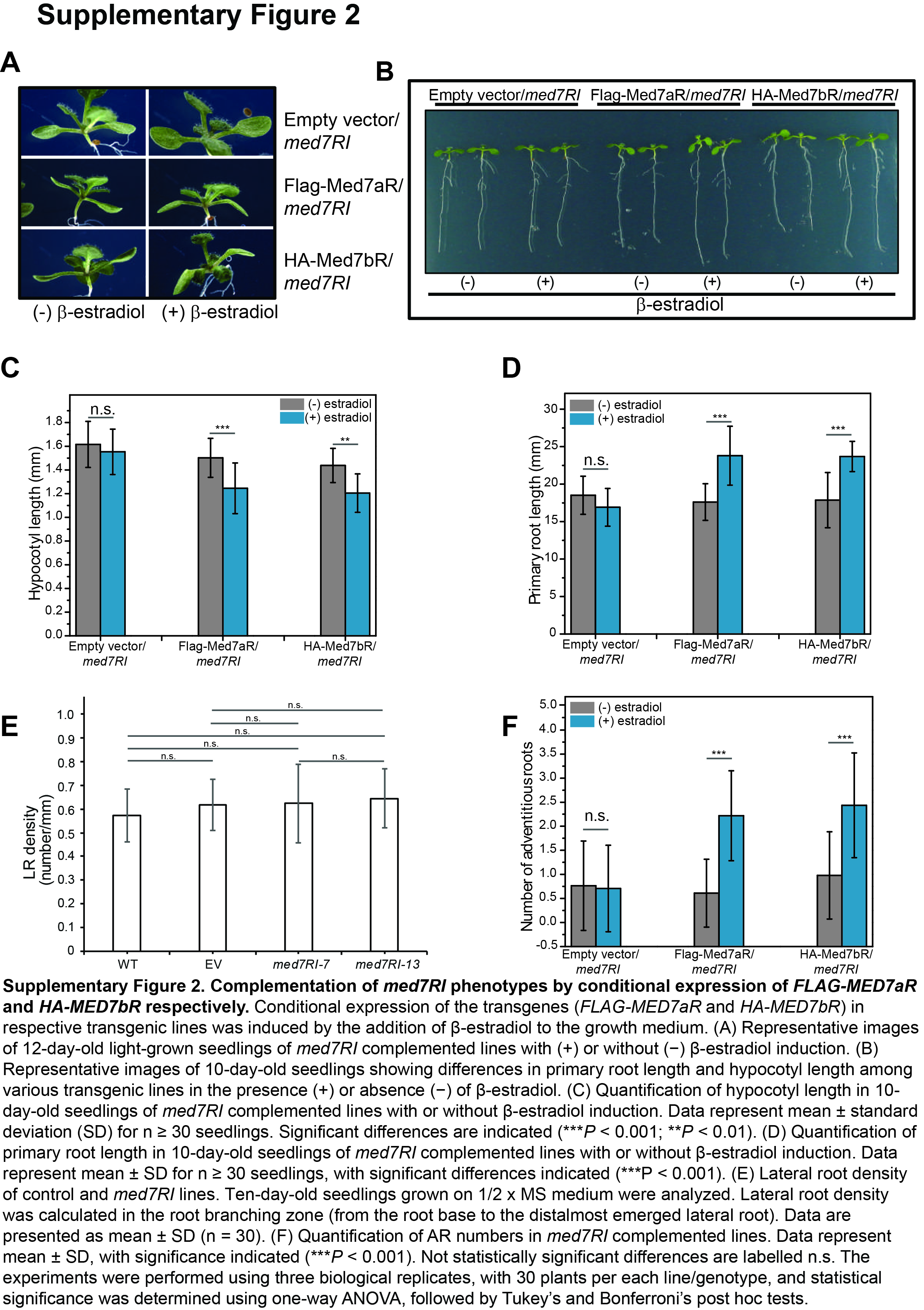

Supplement: Supplementary file 2 [file Image2.jpeg]
